# SERIOUS MENTAL ILLNESS EXACERBATION POST-BEREAVEMENT: A POPULATION-BASED STUDY OF PARTNERS AND ADULT CHILDREN

**DOI:** 10.1093/geroni/igac059.1418

**Published:** 2022-12-20

**Authors:** Djin Tay, Lau Thygesen, Katherine Ornstein

**Affiliations:** University of Utah, Salt Lake City, Utah, United States; University of Southern Denmark, Odense, Syddanmark, Denmark; Johns Hopkins, University, Baltimore, Maryland, United States

## Abstract

The death of a family member may trigger exacerbations among individuals with serious mental illness (SMI). We hypothesized that bereavement would be associated with SMI exacerbations among bereaved partners and adult children diagnosed with SMI. Using linked population-based registries in Denmark, we identified partners and adult children diagnosed with schizophrenia, schizoaffective disorder, bipolar disorder, and major depression in the five years preceding the family member’s death. Generalized estimating equations were used to estimate the odds of SMI exacerbation two years after decedent death. Partners had increased odds of SMI exacerbation at 3 months into bereavement compared to 9-12 months prior to partners’ death (AOR=1.43, [1.13-1.81]). Children with a history of SMI had lower odds of SMI exacerbation in the second year of bereavement. Sociodemographic characteristics and co-occurring alcohol and substance abuse disorders were associated with higher odds of SMI exacerbations. These findings have implications for targeted bereavement support.

